# Evaluation of the combined efficacy of inhibitors of heat shock protein 90 and calcineurin with commonly used antifungals against *Aspergillus*, *Rhizopus*, and *Fusarium isolates*

**DOI:** 10.22034/cmm.2025.345248.1601

**Published:** 2025-04-09

**Authors:** Nuri Kiraz, Sümeyye Şen Kaya, Ayşe Barış, Deniz Turan, Yasemin Öz, İlknur Dağ

**Affiliations:** 1 Istanbul University-Cerrahpasa, Faculty of Medicine, Department of Medical Microbiology, Istanbul, Turkey; 2 University of Health Sciences, Sisli Hamidiye Etfal Training and Research Hospital, Department of Medical Microbiology, Istanbul, Turkey; 3 University of Health Sciences, Haydarpasa Numune Training and Research Hospital, Department of Medical Microbiology, Istanbul, Turkey; 4 Eskisehir Osmangazi University, Faculty of Medicine, Department of Medical Microbiology, Eskisehir, Turkey; 5 Eskisehir Osmangazi University, Vocational Health Services High School, Eskisehir, Turkey

**Keywords:** Antifungal combination, *Aspergillus*, *Fusarium*, Hsp90/calcineurin inhibitors, *Rhizopus*

## Abstract

**Background and Purpose:**

The high morbidity and mortality caused by invasive mold infections require new and effective treatment strategies. Heat shock proteins, which are found in all living organisms, play a role in the homeostatic control of the cell and the stress response mediated by calcineurin. Their release increases especially under stress conditions, and they play a role in ensuring the stability of cellular proteins. Therefore, inhibition of Hsp90 or calcineurin may be an effective method in antifungal therapy. This study aimed to evaluate the *in vitro* activity of four different antifungal agents (caspofungin, amphotericin B, itraconazole, and voriconazole) in combination with fungal stress response regulators, Hsp90 inhibitors, and calcineurin inhibitors, against clinical isolates of *Aspergillus*, *Rhizopus*, and *Fusarium*.

**Materials and Methods:**

In this study, the effectiveness of Hsp90 inhibitors geldanamycin, 17-(allylamino)-17-demethoxygeldanamycin (17-AAG), radicicol, novobiocin (NOV), and calcineurin inhibitors cyclosporine, tacrolimus (TAC), and rapamycin (RAP) combined with common antifungals itraconazole (ITRA), voriconazole (VOR), caspofungin (CAS), and amphotericin B (AMB) were investigated against four *Aspergillus*, three *Rhizopus*, and three *Fusarium* isolates using checkerboard method.

**Results:**

The minimum inhibitory concentration (MIC)/minimum effective concentration (MEC) values of ITRA, VOR, CAS, and AMB were ≤ 0.25, ≤ 0.06-0.125, ≤ 0.03-> 4, and 1-4 µg/mL for *Aspergillus* spp.; 2-8, > 4, > 4, and 2 µg/mL for *Rhizopus* spp.; 8- > 16, 1-4, > 4, and 2-4 µg/mL for *Fusarium* spp., respectively. Although tacrolimus was found to have generally low MIC values (≤0.03 µg/mL) for *Aspergillus* and *Rhizopus* isolates, NOV, and 17-AAG did not exhibit antifungal activity (MICs>128 and ≥16 µg/mL, respectively) against almost all of the isolates. In combination testing against *Aspergillus* and *Rhizopus* spp., synergistic interactions were prevalent (≥75%) for the combinations of ITRA and all inhibitory substances, except for TAC. The effects of CAS and TAC in combination tests were weak. Moreover, synergistic interactions were not frequent in all combinations against *Fusarium* spp. However, antagonistic interaction was observed only in one ITRA and RAP combination throughout this study.

**Conclusion:**

The Hsp90 and calcineurin inhibitors did not have significant antifungal activity alone. Moreover, they did not show a significant antagonistic effect in combination and even increased the efficacy of antifungals at some concentrations.

## Introduction

Invasive mold infections are less common than superficial fungal infections, but their incidence is increasing due to the limited availability of treatment options and the growing problem of resistance [ 1 , 2 ]. *Aspergillus fumigatus*, followed by *Aspergillus flavus* are the most common causes of invasive aspergillosis, which has a high mortality rate. Genera of *Fusarium* and *Rhizopus*, which include species intrinsically resistant to most antifungal agents, are opportunistic fungal pathogens in immunocompromised individuals [ 3 - 5 ]. In particular, *F. oxysporum* is one of the most common species in human fusariosis [ 6 ]. Mucorales infections (mucormycosis) are rare but serious fungal infections with a fulminant course and high mortality rates. Their most common agent is *Rhizopus oryzae*. 

Successful management of these infections relies on early diagnosis and appropriate antifungal therapy. Currently, there are three main groups of antifungal agents for the treatment of invasive fungal infections. However, emerging resistance in *Aspergillus* species and intrinsically antifungal resistance in species of *Rhizopus* and *Fusarium* have further limited antifungal treatment options. Therefore, there is an increasing need to investigate new treatment approaches for the treatment of invasive mold infections [ 7 - 9 ]. 

Heat shock proteins (Hsps), which can be found everywhere in the cell and are classified according to their molecular weight, facilitate the survival of the organism by responding to stress-related changes [ 10 , 11 ]. Hsp90 is a highly conserved molecular chaperone and stimulates the development of drug resistance and antifungal drug tolerance in *Candida* and *Aspergillus* species by multiple molecular mechanisms. Therefore, Hsp90 is necessary for all eukaryotes and regulates the form and function of various client proteins that are key signal transducers. Various animal model studies have reported that the inhibition of Hsp90 function restores the sensitivity in drug-resistant fungal pathogens and prevents the development of drug resistance [ 12 , 13 ]. Hence, targeting a highly conserved protein that plays a vital role in many cellular signaling pathways reduces the resistance to Hsp inhibitors [ 14 ]. Therefore, signal transduction mechanisms, including key secondary messenger calcium, which is important for the adaptation and survival of fungi to the environment, have garnered significant attention. 

Calcineurin is one of the important regulators of intracellular calcium homeostasis in some fungi, and the calcineurin signal cascade is associated with antifungal resistance. A combination of antifungal agents and calcineurin inhibitors has shown inhibitory effects on the growth of drug-resistant fungal strains. It is reported that the combined use of calcineurin inhibitor tacrolimus (FK506) with caspofungin shows a synergistic fungicidal activity against echinocandin-resistant *C. dubliniensis*. In addition, the combination of tacrolimus and azole has shown synergistic antifungal activity against azole-resistant *C. albicans* [ 15 - 17 ]. The present study aimed to evaluate the *in vitro* activity of the combinations of four different antifungals with fungal stress response regulators, Hsp90 inhibitors, and calcineurin inhibitors, against clinical isolates of *Aspergillus*, *Rhizopus* and *Fusarium*.

## Materials and Methods

Isolates and media

In total, 10 clinical mold isolates, namely *Aspergillus fumigatus* (n=3), *A. flavus* (n=1), *Fusarium oxysporum* (n=3), and *Rhizopus oryzae* (n=3) isolated from respiratory and biopsy specimens were included. These isolates have been previously identified by conventional and molecular methods [ 18 ]. *Candida krusei* ATCC 6258 and *C. parapsilosis* ATCC 22019 quality control strains recommended by CLSI M38-A3 were used to determine the antifungal susceptibility of filamentous fungi. In this protocol, the minimum inhibitory concentration (MIC) range of quality control (QC) strains is defined for each drug. Since the accuracy of the applied test is evaluated according to the response of each drug to QC strains, yeast-like fungi can also be used as QC in the susceptibility of filamentous fungi [ 19 , 20 ]. Sabouraud dextrose agar (SDA, Merck, Darmstadt, Germany) was used for the pure culture of isolates, and RPMI 1640 medium (Sigma Chemical Co, St Louis, MO, USA) buffered by MOPS (3-N-morpholinopropanesulfonic acid was used for broth microdilution testing and checkerboard dilution method. Before testing, fresh cultures of all isolates were provided using potato dextrose agar (PDA, Merck, Darmstadt, Germany) at 35 °C.

Antifungal agents and inhibitory substances

Stock solutions of voriconazole (VOR, Sigma Chemical Co, St Louis, MO, USA), itraconazole (ITRA, Sigma), and amphotericin B (AMB, Sigma) were prepared in dimethyl sulfoxide (DMSO) at 1,600 μg/mL concentrations while caspofungin (CAS, Sigma) was prepared in distilled water at 1,600 μg/mL concentrations. Based on our previous studies and the literature data presented below, non-antifungal drug concentrations were determined [ 21 - 24 ]. The Hsp90 inhibitors geldanamycin (GELD), 17-(allylamino)-17-demethoxygeldanamycin (17-AAG), radicicol (RDC), novobiocin (NOV), and calcineurin inhibitors cyclosporine (CICA), tacrolimus (TAC), and rapamycin (RAP) were used as stress response regulators. Stock solutions of TAC, 17-AAG, GELD, RAP (InvivoGen, California, USA), CICA, and RDC (Sigma) were prepared in DMSO at 1,600 µg/mL concentrations. Moreover, NOV (Sigma) was prepared in distilled water at 5,120 μg/mL concentration. All stock solutions were portioned and stored at -70 °C until use.

Antifungal susceptibility testing

Antifungal susceptibility testing was performed by broth microdilution method according to Clinical and Laboratory Standards Institute M38-A3 documents [ 25 - 27 ]. Briefly, conidial inoculum suspensions were prepared from 3-5-day-old sporulated cultures on PDA by using sterile 0.85% saline. The final inoculum concentration was adjusted to 0.4-5 × 10^4^ CFU/mL in RPMI 1640. Sterile, U-bottom, 96-well microplates were used for microdilution testing. Final concentrations of antifungals ranged from 0.0313 to 16 µg/mL for AMB, ITRA, and VOR, and 0.015 - 8 μg/mL for CAS. Final concentrations of inhibitor substances were within the range of 0.03-16 µg/mL for TAC, 17-AAG, GELD, and CICA; 0.008-4 μg/mL for RDC and RAP; and 0.25-128 μg/mL for NOV. The first column, including the drug-free medium, was designed as the growth control wells. The microplates were covered with sterile parafilm and incubated at 35°C. Results were evaluated visually after 24 h incubation for *R. oryzae* and 48 h incubation for other strains. The MIC values were read as the first well in which visible growth was completely inhibited for AMB, ITRA, and VOR. Minimum effective concentration (MEC) value for caspofungin was evaluated microscopically as the lowest drug concentration causing significant shortening and blunting at hyphae. For other substances, the presence of any inhibition in growth (50% or more) was evaluated.

Combination testing: checkerboard method

The concentration ranges in the checkerboard combination method were determined according to MIC results obtained from susceptibility tests; the concentrations ranged from 1/32 to 8x MIC for antifungal agents and from 1/8 to 8x MIC for inhibitor substances. Dilutions of drugs and substances were prepared in RPMI 1640 medium at four times the final concentrations in the microplates. A total of 50 µL of each solution was dispensed, with antifungals placed in the rows and inhibitor substances in the columns of a 96-well microplate. Fungal inoculums were prepared and inoculated to all wells as described above and incubated at 35°C. The results were visually read after 24 h for *R. oryzae* and 48 h for other strains. When the absence of adequate growth in the growth control well was observed, the incubation was extended more than 24 h [ 28 ]. In order to evaluate the activity of the combinations, the fractional inhibitor concentration index (FICI) was calculated as follows: MICA in combination/MICA alone + MICB in combination/MICB alone. Drug interactions were defined as synergistic (FICI≤0.5), no interaction/indifference (0.5<FICI < 4), or antagonistic (FICI≥4), [ 29 , 13 ]. Off-scale MIC values were converted to the next highest two-fold concentration.

## Results

The MIC/MEC results of drugs and substances for each isolate are presented in Table 1. The lowest MIC/MEC values for antifungals were exhibited against *Aspergillus* spp. While the lowest MIC ranges for isolates of *R. oryzae* were observed with AMB (2.0 µg/mL) as expected, the highest MIC/MEC ranges for all drugs were detected against *F. oxysporum*. The MIC values of the reference isolates were acceptable. Among the inhibitors, the most apparent effect was observed in TAC, especially against *Aspergillus* and *Rhizopus* isolates (≤0.03 µg/mL). Although RAP and CICA had MIC values against almost all isolates, GELD, 17-AAG, and RDC had limited activity, and NOV did not exhibit any antifungal activity. 

**Table 1:** *In vitro* antifungal activities of antifungals and Hsp90/calcineurin inhibitors used alone against clinical isolates of Aspergillus, Rhizopus, and Fusarium

Isolates	MIC/MEC values of drugs (µg/mL)				MIC values of inhibitory substances (µg/mL)						
	ITRA	VOR	CAS	AMB	RAP	TAC	CICA	GELD	RDC	NOV	AAG
*A. fumigatus* 1342	≤0.25	≤0.06	≤0.03	2	1	≤0.03	1	16	>4	>128	>16
*A. fumigatus* 4373	≤0.25	≤0.06	>4	1	1	≤0.03	2	16	>4	>128	>16
*A. fumigatus* 1345	≤0.25	≤0.06	≤0.03	4	>4	>16	4	>16	4	>128	>16
*A. flavus* 1343	≤0.25	0.125	≤0.03	2	1	≤0.03	>16	16	>4	>128	>16
*R.oryzae* YDH	8	>4	>4	2	>4	≤0.03	4	16	4	>128	16
*R.oryzae* 1373	4	>4	>4	2	4	≤0.03	1	>16	4	>128	>16
*R.oryzae* 8204	2	>4	>4	2	0.5	≤0.03	2	8	0.125	>128	4
*F.oxysporum*-5028	>16	4	>4	2	2	4	>16	8	>4	>128	>16
*F.oxysporum* 1384	>16	1	>4	4	64	8	>16	8	>4	>128	>16
*F.oxysporum* 6683	8	1	>4	2	1	>16	8	16	4	>128	>16

MIC: minimum inhibitory concentration, MEC: minimum effective concentration, ITRA, itraconazole; VOR, voriconazole; CAS, caspofungin; AMB, amphotericin B; RAP, rapamycin; TAC, tacrolimus; CICA, cyclosporin A; GEL, geldanamycin; RDC, radicicol; NOV, novobiocin; AAG, 17-allylamino-17-demethoxygeldanamycin.

The checkerboard testing results are summarized in Table 2 and Supplementary Table 1. A total of 280 combination tests (28 different combination tests for each isolate) were studied. Antagonistic interaction was observed for only one combination (RAP+ITRA) against *A. fumigatus*-4373 isolate. Moreover, 187 (67%) combinations resulted in indifference and 92 (33%) showed synergistic interaction. Especially, the combination of RAP + ITRA resulted in synergistic interaction against eight isolates (Supplementary Table 1). In general, the most promising results were observed for the combinations of inhibitory substances with ITRA, followed by their combinations with AMB (Table 2). While MICs of ITRA alone were high (2->16 µg/mL) for almost all isolates (except *Aspergillus* spp.), significant decreases were observed in combination with inhibitory substances (except for Fusarium spp.), often resulting in synergy (Supplementary Table 1). It should be mentioned that similar results were obtained for AMB. However, synergistic interactions were not apparent for the combinations of inhibitory substances with CAS. A noteworthy observation was that while CAS MICs were > 4 µg/mL for *Rhizopus* oryzae isolates in combination with TAC, it resulted in synergistic interactions for all three isolates.

**Table 2:** Rate of isolates resulting in synergistic interaction according to checkerboard combination tests (number of isolates resulting in synergistic interaction/total number of isolates).

Inhibitory substances	*Aspergillus* species (n=4)				*Rhizopus oryzae* (n=3)				*Fusarium oxysporum* (n=3)			
	ITRA	VOR	CAS	AMB	ITRA	VOR	CAS	AMB	ITRA	VOR	CAS	AMB
RAP	0.75	0.25	0	0.50	1.00	0.33	0	0.66	0.66	0.33	0	0
TAC	0	0	0	0.25	0	0	1.00	0.33	0	0.33	0.33	0
CICA	0.75	0.25	0	0.25	0.66	0	0	1.00	0	0.33	0.33	0.33
GELD	1.00	0.50	0	1.00	0.66	0.33	0	0.66	0	0.33	0.33	0.33
RDC	0.75	0.25	0	0.5	1.00	0	0	0.33	0	0	0.33	0
NOV	0.75	0.50	0	0.75	0.33	0	0	0.33	0.33	0.33	0.33	0
17-AAG	1.00	0.50	0.25	0.25	0.66	0	0	0.66	0	0.33	0	0.66

ITRA: itraconazole; VOR: voriconazole; CAS: caspofungin; AMB: amphotericin B; RAP: rapamycin; TAC: tacrolimus; CICA: cyclosporin A; GEL: geldanamycin; RDC: radicicol; NOV: novobiocin; 17-AAG: 17-allylamino-17-demethoxygeldanamycin.

In addition, the rate of synergistic interactions for all combinations was the lowest against *F. oxysporum* isolates. 

## Discussion

Increasing frequency of invasive fungal infections and the emerging problem of antifungal resistance have increased interest in the studies on new treatment alternatives and combination therapy [ 30 , 31 ]. In particular, combinations of antifungal agents with Hsp and calcineurin inhibitors are promising. In this study, combinations of some Hsp90 calcineurin inhibitors with commonly used antifungals were evaluated against *Aspergillus*, *Rhizopus*, and *Fusarium* isolates. 

In literature, it is reported that compounds acting on Hsp90 and calcineurin have different activities on different fungal species. Lamoth et al. investigated the antifungal activity of some components targeting the Hsp90-calcineurin pathway against 62 mold species. In their study, TAC showed variable activities on different *Aspergillus* species (MIC range: 0.025-0.4 μg/mL for *A. fumigatus* and *A. flavus* isolates) and was effective on *Mucor* species (MIC range: 0.012->0.4 μg/mL) while GELD was found to be effective on *Fusarium* species [ 32 ]. Similarly, in the present study, the most prominent effect was obtained with TAC. This effect is very pronounced, especially in *Aspergillus* and *Rhizopus* isolates and data are consistent with literature. 

According to the combination test results in the present study, synergistic interaction was frequently observed in the combinations of GELD and 17-AAG with ITRA or AMB, especially against *Aspergillus* and *Rhizopus* isolates. In addition, CICA-AMB, TAC-CAS, and RDC-ITRA showed synergistic interaction against all *R. oryzae* isolates, and no antagonism was found in any of the combinations. These results are in accordance with the literature [ 33 ]. In addition, a synergistic interaction was detected against all *Aspergillus* isolates (n=4) in the combination of GELD and AMB, GELD and ITRA, as well as 17-AAG and ITRA. However, no combinations showing synergistic interactions against all *Fusarium* isolates were observed. Although it has been reported in the literature that RAP shows synergy with various antifungals, this combination was generally found to be indifferent in the present study. Moreover, the RAP-ITRA combination for one isolate (*A. flavus*) resulted in an antagonistic interaction. 

The main goal of echinocandin drugs is to disrupt the stability of the cell wall. Especially in *A. fumigatus*, it is reported that calcineurin inhibitors increase echinocandin activity and transform the fungistatic activity of caspofungin into fungicidal activity. In addition, according to previous studies, these inhibitors show potent activity against azole and echinocandin-resistant *A. fumigatus* isolates [ 34 , 35 ]. These findings are consistent with those of the present study as CAS-TAC resulted in synergy in all isolates. These data may provide insights for further studies.

The RDC also binds to Hsp90 with strong affinity and inhibits ATP-dependent chaperone function. In the present study, when RDC was used alone, it showed the most significant antifungal effect against *R. oryzae* isolates (MIC range: 0.125-4 µg/ml). There was no significant antifungal effect against other mold isolates at the studied concentrations. Although the majority of combinations of RDC with antifungal agents showed indifferent efficacy, it is noteworthy that none of the 40 evaluated combinations showed antagonism. It was also promising that all combinations of RDC+ITRA, two combinations of RDC+AMB, and one combination of VOR+RDC showed synergy against isolates of *A. fumigatus* and *R. oryzae*.

The NOV is an effective coumarin antibiotic against Gram-positive bacteria, and it binds to the C terminal of Hsp90, causing these proteins to degrade [ 36 ]. As with the other Hsp90 inhibitors, it is thought that NOV can enhance the effectiveness of antifungals. Although the antifungal activity of a chemical derivative of NOV alone, which is 1,000 times more potent, has been demonstrated [ 37 ], the contribution of the original NOV to antifungals has not been fully elucidated yet. In the present study, NOV alone did not show any activity against isolates (MIC>128 µg/ml for all isolates). However, synergistic interactions observed in NOV-antifungal combinations were most prominent, especially in NOV-ITRA combinations.

Substances that act through the inhibition of Hsp90 and calcineurin enhance the effectiveness of antifungals, possibly by reducing the stress response that occurs in fungal cells exposed to antifungal agents, thereby decreasing resistance to antifungals. Therefore, they can create a promising combination strategy against mold isolates. However, since these inhibitors do not have fungal specificity, their use as antifungals is limited. 

## Conclusion

Considering all 28 combination tests for each isolate, a negligible number of antagonistic interactions were observed. The synergistic interaction obtained for many combinations may be promising in the management of invasive fungal infections, especially considering the known resistance issues for the fungi included in this study. The most important limitation of this study was the small number of isolates. However, due to the inclusion of a large number of inhibitory substances, the obtained results can serve as a guide for planning future studies.
